# Visualization of energy‐based device‐induced thermal tissue alterations using bimodal ex‐vivo confocal microscopy with digital staining. A proof‐of‐concept study

**DOI:** 10.1111/srt.13155

**Published:** 2022-04-12

**Authors:** Gabriella Fredman, Rikke Louise Christensen, Vinzent Kevin Ortner, Merete Haedersdal

**Affiliations:** ^1^ Department of Dermatology University Hospitals of Copenhagen, Bispebjerg and Frederiksberg Copenhagen Denmark

**Keywords:** acridine orange, coagulation zone, ex‐vivo confocal microscopy, fractional CO_2_‐laser, microscopic ablation zone, radiofrequency microneedling

## Abstract

**Background:**

Ex‐vivo confocal microscopy (EVCM) enables examination of tissue alterations immediately after treatment with energy‐based devices (EBDs). This proof‐of‐concept study aimed to describe EBD‐induced tissue effects in ex‐vivo porcine skin after treatment with microneedle radiofrequency (MNRF) and ablative fractional CO_2_‐laser (AFL) using EVCM.

**Materials and Methods:**

Ex‐vivo porcine skin was treated with MNRF and AFL. Three cryosections from each intervention were stained with acridine orange (AO) and scanned with EVCM. Reflectance confocal microscopy (RCM, 638 nm) and fluorescence confocal microscopy (FCM, 488 nm) images were captured and evaluated individually, after image fusion, and after digital hematoxylin and eosin (H&E) staining.

**Results:**

Bimodal EVCM was able to visualize EBD‐induced thermal alterations in porcine skin. In RCM mode, the full width and depth of the vertically aligned microscopic treatment zones (MTZs) were displayed with clear demarcation to surrounding intact skin. In FCM mode, the ablation of the epidermis after AFL was prominent in contrast with the almost intact epidermis observed in MNRF treated skin. In fusion mode, fluorescence signal from AO marked the surrounding coagulation zone (CZ) from both interventions, with enhanced discrimination between ablation and coagulation. Digitally H&E‐stained images closely resembled conventional histopathology but proved superior in terms of visualization of the CZ.

**Conclusion:**

Bimodal EVCM with digital H&E‐staining facilitates the identification and qualitative evaluation of thermal alterations induced by treatment with EBD. By providing high‐resolution images comparable to standard histology, EVCM is a useful tool in the research and development of EBD to visualize and evaluate device‐tissue interactions.

## 1 INTRODUCTION

Energy‐based device (EBD)‐induced thermal effects in skin have traditionally been evaluated by histological examination.^1^ However, tissue processing prior to histology introduces artefacts that may interfere with image interpretation. To overcome this challenge, ex‐vivo confocal microscopy (EVCM) is emerging as an alternative to conventional histology by permitting real‐time imaging of excised tissue at the cellular level to examine tissue alterations directly after treatment.^2^ With EVCM, exogenous fluorescence‐contrast agents for nuclear staining and endogenous reflectance contrast from dermal collagen and cytoplasm can be combined into digitally stained images that mimic readily interpretable conventional hematoxylin and eosin (H&E) staining.^3^

The ablative fractional CO_2_‐laser (AFL) is a medical device for various treatment indications such as photodamage, scars, and striae.^4^, ^5^, ^6^, ^7^, ^8^, ^9^ By delivering fractionated light in narrow columns to the skin, numerous microscopic treatment zones (MTZs) are created, inducing epidermal reepithelization and dermal remodelling of the altered tissue.^4^, ^5^, ^6^, ^7^, ^8^, ^9^ Depending on the laser settings, the depth and width of the vertical microscopic ablation zone (MAZ) and the dimensions of the surrounding coagulation zone (CZ) can be adjusted.^10^, ^11^, ^12^ Used for same indications as the AFL, radiofrequency microneedling (MNRF) is increasingly applied in the clinical setting to avoid some of the side effects associated with ablative lasers.^13^ By utilizing the intrinsic resistance of tissue, radiofrequency converts electrical current into thermal energy. Through microneedles, thermal energy is transmitted to a pre‐targeted depth where it causes coagulation and collagen denaturation inducing the tissue remodelling process.^13^, ^14^, ^15^

This study aimed to explore the utility of EVCM for qualitative evaluation of acute thermal alterations from MNRF and AFL in ex‐vivo pig skin. For nuclear staining and demarcation of the CZ, the tissue was stained with the fluorescent probe acridine orange (AO).^16^, ^17^ In addition, the performance of digital H&E staining was compared to conventional histopathology.

## 2 METHODS AND MATERIALS

### 2.1 Study design

In this proof‐of‐concept study, immediate tissue alterations in ex‐vivo porcine skin treated with MNRF and AFL were investigated with EVCM. Cryosections of MNRF‐ and AFL‐treated skin were qualitatively assessed for thermal ablation and coagulation. The study was conducted at the Department of Dermatology, Copenhagen University Hospital, Bispebjerg, Copenhagen, Denmark.

### 2.2 Tissue preparation

Full‐thickness skin was collected from the flank of a female pig (mixed race, 34 kg). Hair and subcutaneous fat were removed and skin with visible signs of damage was discarded before storage at −80˚C up to 12 weeks. Skin samples were thawed to room temperature prior to treatment.

Before EVCM imaging, 8 mm punch biopsies from treated skin were sectioned into 100 μm thick slices with a cryostat and freezing medium (Tissue‐Tek® O.C.T.™ Compound; Sakura Finetek Europe BV, Alphen, NL).^18^ EVCM was applied on cryosections to avoid the artifacts that commonly appear when examining full thickness biopsies due to uneven pressure and folding of tissue.

Three cryosections of treated skin from each intervention were examined with the EVCM.

### 2.3 Microneedle radiofrequency treatment

MNRF treatment was performed with a fractional MNRF (Lutronic Genius®) delivering energy of 133.3 mJ/pin at a power level of 48 W with a pulse duration of 100 ms through insulated 1.5‐mm long microneedles.

### 2.4 Ablative fractional CO_2_‐laser treatment

AFL treatment was performed with a 10,600 nm fractional CO_2_‐laser (UltraPulse®, Deep Fx handpiece, Lumenis Inc., Santa Clara, CA) delivering single passes of 50 mJ at 15% density.

### 2.5 Ex‐vivo confocal microscopy

Each tissue specimen was rinsed in sodium chloride before being submerged into a solution of AO for 10 seconds. A shorter staining duration than established in previous studies was chosen due to the use of 100 μm tissue sections instead of full‐thickness skin.^19^ After staining, excess AO was removed by soaking the tissue specimen again in sodium chloride for 10 seconds. Samples were mounted between two glass slides before imaging with a commercially available EVCM (Vivascope® 2500, MAVIG GmbH, Munich, Germany). The EVCM uses two laser excitation wavelengths to capture images in reflectance (638 nm) and fluorescent mode (488 nm).^20^ By binding to the DNA and cytoplasmic RNA in cells, an increased nuclei‐to‐dermis contrast is provided when AO‐stained tissue (Exλ = 460−500 nm; Emλ = 526−650 nm) is illuminated with the 488‐nm wavelength.^21^ As only weak fluorescence is collected from the dermis and subcutaneous fat, a 1000‐fold enhanced contrast of the epidermis and adnexal structures to cytoplasm and sub‐epidermal structures is obtained.^3^, ^21^

### 2.6 Image analysis

Thermal alterations were qualitatively evaluated in reflectance confocal microscopy (RCM), fluorescence confocal microscopy (FCM) and in combined RCM/FCM images. With computer algorithms, confocal scanning in overlayed reflectance (RCM) and fluorescent (FCM) mode can generate pink‐ and purple digital H&E staining, resembling the conventional histology.^19^ The utility of digitally H&E‐stained confocal scans was compared to conventional histopathological slides.

Images were processed in Fiji ImageJ® for pseudocoloring and brightness and contrast adjustments.

### 2.7 Histological examination

To assess the feasibility of EVCM in evaluating ablative and thermal tissue alterations from MNRF and AFL, one biopsy for each intervention was cut into 10‐μm thick slices and stained using conventional H&E. Histology images were captured using a digital slidescanner (MoticEasyScan®, Motic Incorporation Ltd.)

## 3 RESULTS

**FIGURE 1:**
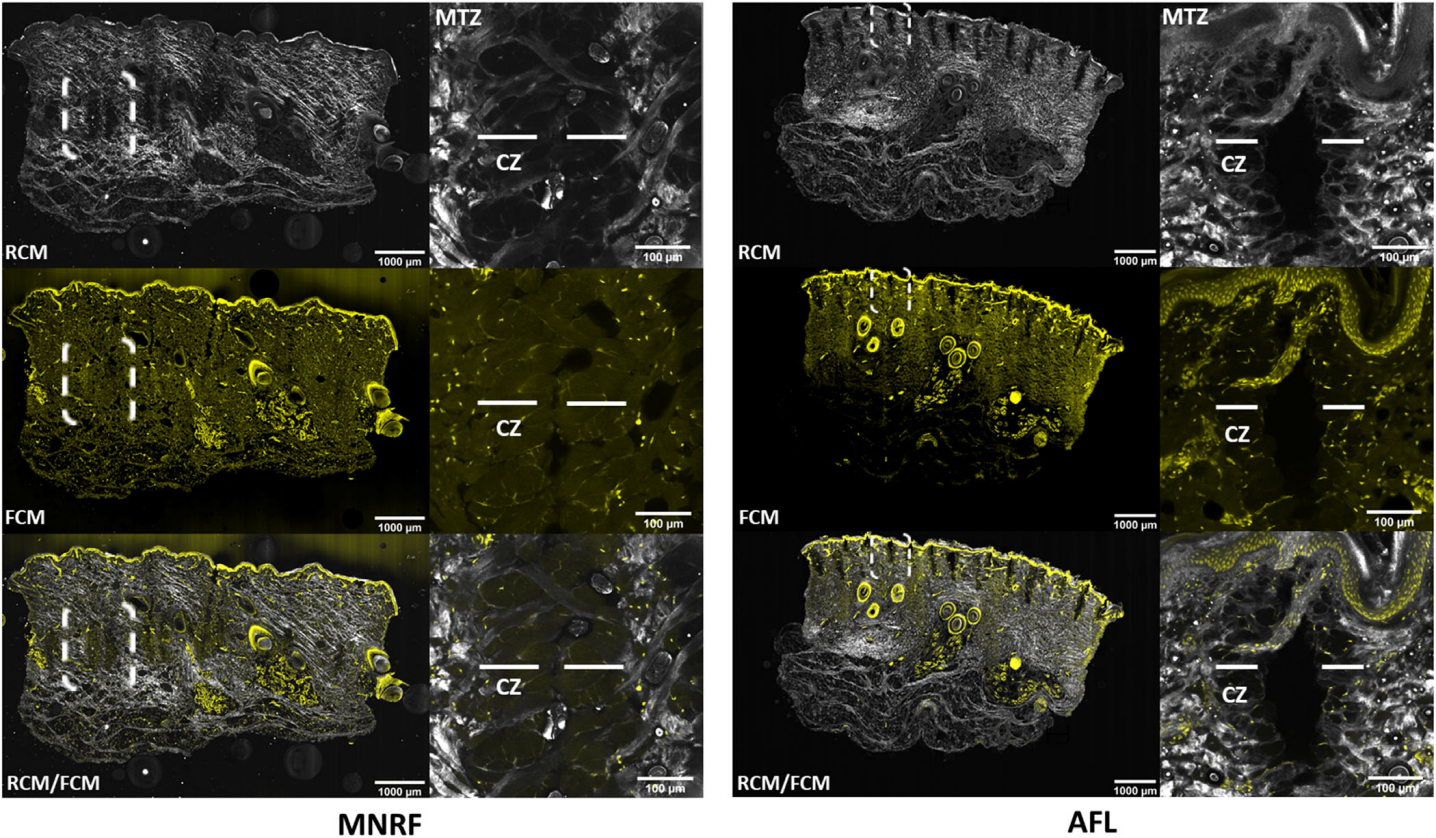
Thermal alterations after radiofrequency microneedling (MNRF) and ablative fractional CO_2_‐laser (AFL) in reflectance, fluorescence, and fusion mode. In the reflectance confocal microcopy (RCM, 638 nm) images, the full width and depth of the microscopic treatment zones (MTZs) after MNRF and AFL are visualized (annotated by white dashed lines). The coagulation zone (CZ) (annotated by white lines) of the MTZ is barely visible, while the ablative zone in RCM images appears larger than in fluorescence confocal microscopy (FCM, 488 nm) images. In the FCM images, central loss of substance in the narrow ablative zone of the MTZ´ (annotated by white dashed lines) is observed as black channels while the surrounding CZ is labelled by acridine orange (AO) (marked by white lines). In combined FCM and RCM mode, discrimination between ablation and coagulation of the MTZ´ is enhanced.

**FIGURE 2:**
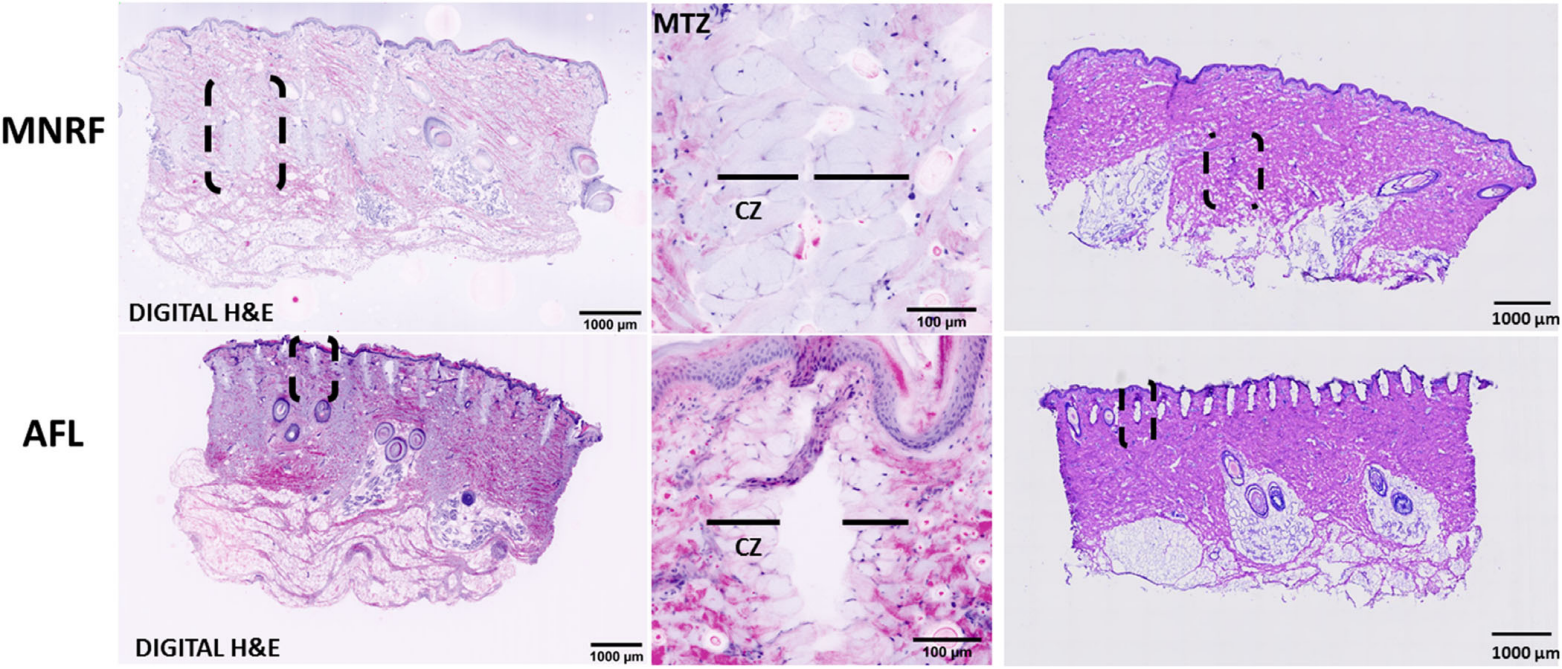
Thermal alterations after radiofrequency microneedling (MNRF) and ablative fractional CO_2_‐laser (AFL) in digital hematoxylin and eosin (H&E) staining compared to histopathology. In digital H&E mode, the vertically aligned microscopic treatment zones (MTZs) from MNRF and AFL treatment (black dashed lines) within the dermis appear with clear visualization of the coagulation zone (CZ) from diffusion of acridine orange (AO) staining, contrasting the histopathological slides where the CZ is barely identifiable. In digital H&E mode of an individual MTZ, the narrow ablative zone is surrounded by the CZ (marked with black lines) stained light purple from diffusion of AO. In the histopathological slides, the MTZ´ from MNRF and AFL are marked by black dashed lines.

Overview images of the tissue sections allowed identification of MTZ´ from MNRF and AFL before detailed analysis in single mode RCM and FCM, as well as in fusion mode and finally with the digital H&E staining. The confocal microscopy findings are presented in Figures 1 and 2 and Table 1, and in the following subsections.

**TABLE 1:** A summary of confocal microscopy features accomplished by RCM, FCM, digital H&E staining as compared to each other and conventional histology

	Ex‐vivo confocal Microscopy			Histology
	RCM (638 nm)	FCM (488 nm)	Digital H&E staining (RCM 638 + FCM 488 nm)	H&E staining
Epidermis				
Cell cytoplasm	+++	+	+++	+++
Cell nuclei	+	+++	+++	+++
Thermal ablation	+++	+++	+++	+++
Thermal coagulation	++	+++	+++	+
Dermis				
Thin collagen fibers	+++	+	+++	+++
Thick collagen bundles	+++	+	+++	+++
Thermal ablation	+++	+++	+++	+++
Thermal coagulation	++	+++	+++	+
Sebaceous/eccrine glands	+	+++	+++	+++
Hair follicles	+	+++	+++	+++
Subcutis				
Thin collagen fibers	+++	+	+++	+++
Adipocytes	++	+	+++	+++

Abbreviations: EVCM, ex‐vivo confocal microscopy; FCM, fluorescence confocal microscopy; H&E, hematoxylin and eosin; nm: nanometer, RCM, reflectance confocal microscopy;.

+++ = Excellent visualization.

++ = Good visualization.

+ = Moderate visualization.

### 3.1 Tissue alterations in reflectance mode

In RCM mode, the hyperreflective dermal collagen presents as a reticulated network of gray, thin fibers and thick bundles of collagen (Figure 1). In MNRF treated skin, the MTZ´ are located in the mid‐deep dermis, whereas AFL‐induced MTZ´ are found in the superficial layers of the dermis. The full width and depth of the vertically aligned MTZ´ after each treatment modality are well demarcated from the surrounding intact skin. The ablation zones of the MTZ´ are observed as black channels, with only weak signal from the barely visible surrounding CZ.

### 3.2 Tissue alterations in fluorescence mode

In FCM mode, the cells of the epidermis and hair follicles are prominent with strong fluorescence signal from AO staining of nucleic acid (Table 1). Compared to the almost intact epidermis observed in MNRF treated skin, ablation of the epidermis after AFL is prominent. The CZ that surrounds the ablation zone is stained by AO but exhibits weaker fluorescence signal than the nucleus‐rich structures.

### 3.3 Bimodal assessment of tissue alterations compared to conventional histology

In fusion mode, the discrimination between ablation and coagulation from both interventions is enhanced. In MNRF treated skin, the ablation zone is narrow with marked coagulation of nearly the entire cross‐section of the MTZ´ (Figure 1). In contrast to the alterations observed after MNRF treatment, the ablation from AFL is more pronounced, stretching vertically from epidermis to the upper part of the dermis, but with comparatively less coagulation. By switching to digital H&E staining, the skin structures morphologically resemble those observed in conventional histopathology (Figure 2). The epidermis and hair follicles are well‐defined, with purple‐stained nuclei of the keratinocytes and sebocytes (Table 1). The bright purple nuclear staining of the adipocytes is in sharp contrast to the pink staining of the connective tissue, thereby improving the visualization of the subcutaneous fat. The reticular fibers and collagen bundles of the dermis are well‐defined and discriminable as they are stained pink similar to conventional H&E staining. Within the dermis, the vertically aligned MTZ´ from MNRF and AFL treatment appear with clear visualization of the CZ from diffusion of AO staining, contrasting the histopathological slides where the CZ is barely identifiable (Figure 2).

## 4 DISCUSSION

In this proof‐of concept study, EVCM was applied for qualitative evaluation of alterations in ex‐vivo porcine skin after exposure to MNRF and AFL. The digital H&E staining provided clear distinction between ablation and coagulation of the MTZ. The demarcation of the thermal alterations was best visualized in the RCM mode, due to the bright contrast of the collagen of adjacent, untreated skin. In the FCM mode, strong fluorescence signal from AO clearly marked the cells of the epidermis and hair follicles and highlighted the epidermal ablation (Table 1).

In the recent years, studies of EVCM have explored the use of digital staining to simulate conventional H&E histology.^2^ While several studies have focused on confocal‐histological correlations, the clinical application of EVCM has mainly proven useful for margin delineation of skin cancer prior to Mohs surgery.^2^ The digitally H&E‐stained confocal scans provide intuitive comparison to standard histology and may substitute histological examination in cases where quick evaluation of tissue is desired.^3^, ^19^ To enhance a broader usage of EVCM in research and for possible clinical indications, recent studies have investigated the use of fluorescent‐labelled antibodies to increase the diagnostic accuracy of EVCM in inflammatory skin diseases with immune depositions such as autoimmune blistering diseases, cutaneous vasculitis, cutaneous lupus and lichen planus.^18^, ^22^, ^23^, ^24^, ^25^ Additional staining methods have been tested to enhance the contrast of different cell structures without altering the tissue prior to histological examination.^2^ Prior to conventional histological examination, tissue preparation and paraffin embedding are required but can lead to tissue shrinkage that might compromise qualitative evaluation of tissue. Since EVCM enables examination of fresh tissue, a more accurate evaluation of the immediate thermal effects after EBD is therefore obtained. As such, EVCM may be useful for initial quick assessment of immediate EBD‐induced thermal changes in clinical research projects and potentially in the clinical setting as a screening tool prior to conventional histological analysis.

In contrast to in‐vivo RCM, the EVCM enables vertical examination of tissue and therefore a more accurate estimate of the dimensions of the MTZ´ generated by MNRF and AFL. Furthermore, it allows the addition of the digital H&E staining which, in our study, proved superior to confocal scanning in the single mode RCM‐ and FCM gray‐scale contrast in terms of discrimination between thermal ablation and the CZ (Table 1). The EVCM technique is based on refraction of light for image generation in RCM and on fluorescence excitation for FCM.^3^ In digitally H&E‐stained images as well as in FCM, the AO staining highlighted the nuclei‐rich structures. Additionally, the CZ was visualized due to accumulation of AO to the surrounding tissue cuff. In RCM, the bright contrast from collagen and cytoplasm and the sparse contrast from cell nuclei visualized the EBD‐induced thermal changes with sharp demarcation to adjacent, untreated skin. In digitally H&E‐stained images, the purple AO labeling of the cell nuclei corresponds to hematoxylin on conventional histopathological slides while the pink staining mimics eosin.^3^

MNRF has become a viable option for treatment of acne, acne scars, striae, and for skin rejuvenation.^26^ By combining microneedles with radiofrequency, dermal heating is improved to the critical level of 65–70˚C—necessary for coagulation and collagen denaturation, but with less epidermal heating compared to AFL.^13^ Contrary to MNRF treatment and as observed in our study, AFL induces thermal ablation of the epidermis which provides longer downtime and a higher rate of side effects especially for darker skin types.^13^ Characterization of the acute thermal injury is therefore essential for proper selection of treatment device based on either skin type or whether the treatment purpose is epidermal reepithelization (photodamage, skin rejuvenation)^6^ or dermal remodeling (scars, keloids, wrinkles).^27^ By adjusting device settings, different outcomes can be achieved depending on the dimensions of the MTZ´ and the proportion between thermal ablation and coagulation.^1^, ^10^, ^11^ For this purpose, the EVCM may be useful in preclinical studies of EBD, to gain basic information of tissue interaction at different settings directly after treatment.

The main limitations of our study are the small sample size and lack of multiple EBD settings that precluded a quantitative evaluation of tissue changes. However, the study was a proof‐of‐concept trial aimed for initial evaluation of the ability of EVCM to visualize acute EBD‐induced thermal changes with focus on the applicability of the digital H&E staining. To gain value as a useful tool in preclinical and clinical studies of EBD, however, multiple EBD settings should be tested and quantified in vivo. Additional consideration is the fragility of unfixed tissue specimens and differences in pressure of tissue when mounted between the microscope slides.^2^, ^18^, ^19^, ^20^, ^28^ Careful handling and optimization of tissue flattening is therefore crucial for future EVCM investigations of EBD‐induced thermal alterations. Based on our experience and to achieve high‐quality images, we applied the EVCM on cryosectioned tissue to avoid the artifacts that commonly appear due to uneven pressure and epidermal folding of full‐thickness skin. However, examination of full‐thickness skin is possible and more time‐efficient than cryosectioned tissue and should therefore be explored in future EVCM studies of EBD.

In conclusion, our study demonstrates the ability of EVCM to visualize acute thermal alterations in ex‐vivo pig skin directly after treatment with MNRF and AFL. Compared to histopathology, confocal scanning with digital H&E staining enables clear discrimination between thermal ablation and coagulation and may be a helpful tool for the development of EBD.
